# Supplementary material for: A Scalable MNase-seq Framework for Reproducible Nucleosome Profiling across Pluripotent Stem Cell and Cardiomyocyte Models
Source: Comput Struct Biotechnol J. 2026 Aug 5;35(1):0204. doi: 10.34133/csbj.0204 (PMC13438036; doi:10.34133/csbj.0204)
Supplement: Supplementary file 1 [file csbj.0204.f1.zip › Supplementary_Table_SpikeIn.docx]

**Supplementary Table S1. Yeast spike-in recovery across the MNase digestion titration in independently sequenced low- and high-depth libraries**

Purified yeast MNase-derived spike-in DNA, generated using the same purification reagents, kits, and buffers as the hiPSC MNase-derived DNA, was combined with purified hiPSC MNase-derived DNA at a fixed ratio (0.5 ng yeast DNA per 10 ng hiPSC DNA, ~5% of total mass) immediately prior to library preparation, with both DNAs undergoing identical library conversion steps (end repair, adapter ligation, PCR amplification, SPRI bead-based purification). Yeast spike-in alignment statistics are reported for all five MNase concentrations (0.5-10 U) in hiPSCs, in both the low-input (50bp paired-end) and high-depth (150bp paired-end) sequencing libraries. Yeast % (of total reads) is calculated as yeast-unique / total sequenced reads per condition. Deviation from 5% target expresses the difference, in percentage points (pp), between the observed yeast fraction and the nominal 5% mixing ratio.

***Supplementary Table S1. Yeast spike-in alignment statistics across the MNase titration, low- and high-depth libraries.***

| **Library** | **MNase (U)** | **Total reads** | **Yeast unique** | **Yeast multi** | **Human unique** | **Human multi** | **Unaligned** | **Yeast % (of total reads)** | **Deviation from 5% target (pp)** |
| --- | --- | --- | --- | --- | --- | --- | --- | --- | --- |
| Low-depth (50bp PE) | 0.5 | 7,659,348 | 468,926 | 83,534 | 4,631,323 | 966,712 | 1,508,853 | 6.1% | +1.1 |
| Low-depth (50bp PE) | 1 | 8,408,494 | 309,361 | 53,904 | 5,555,234 | 1,270,736 | 1,219,259 | 3.7% | -1.3 |
| Low-depth (50bp PE) | 3 | 5,930,979 | 250,082 | 46,205 | 3,544,284 | 1,135,266 | 955,142 | 4.2% | -0.8 |
| Low-depth (50bp PE) | 6 | 5,935,171 | 205,618 | 36,997 | 3,619,006 | 1,289,608 | 783,942 | 3.5% | -1.5 |
| Low-depth (50bp PE) | 10 | 7,814,607 | 326,246 | 65,684 | 4,371,662 | 2,245,629 | 805,386 | 4.2% | -0.8 |
| High-depth (150bp PE) | 0.5 | 79,128,551 | 5,321,683 | 1,241,234 | 37,136,223 | 18,217,068 | 17,212,343 | 6.7% | +1.7 |
| High-depth (150bp PE) | 1 | 87,859,308 | 3,658,675 | 932,098 | 45,224,353 | 24,822,824 | 13,221,358 | 4.2% | -0.8 |
| High-depth (150bp PE) | 3 | 68,310,881 | 3,488,667 | 920,108 | 32,769,121 | 24,409,030 | 6,723,955 | 5.1% | +0.1 |
| High-depth (150bp PE) | 6 | 65,362,382 | 2,676,382 | 737,099 | 31,672,799 | 25,453,720 | 4,822,382 | 4.1% | -0.9 |
| High-depth (150bp PE) | 10 | 84,423,544 | 4,045,979 | 1,150,350 | 38,238,494 | 37,465,264 | 3,523,457 | 4.8% | -0.2 |

Across all ten independently sequenced libraries (five MNase concentrations x two sequencing depths), the yeast spike-in fraction remained within ±1.7 percentage points of the nominal 5% mixing ratio (range 3.5-6.7%; overall mean 4.7%), with no consistent monotonic relationship to MNase concentration. As the purified yeast spike-in DNA is added at a fixed ratio downstream of MNase digestion, rather than co-digested with the experimental sample, it is not itself subject to the digestion conditions being titrated; the modest condition-to-condition variability observed instead reflects normal inter-library technical variation introduced during spike-in aliquoting, mixing, and library conversion. The consistency of the observed yeast fraction with the intended 5% target, and its concordance across two independently prepared and sequenced library sets differing in both depth and read length, demonstrates the reliability of the yeast spike-in as a normalization control across the full range of digestion conditions tested in this study.

*Note: 'pp' denotes percentage points (the absolute difference between two percentages), distinct from a relative percentage change.*
