# Supplementary material for: A Scalable MNase-seq Framework for Reproducible Nucleosome Profiling across Pluripotent Stem Cell and Cardiomyocyte Models
Source: Comput Struct Biotechnol J. 2026 Aug 5;35(1):0204. doi: 10.34133/csbj.0204 (PMC13438036; doi:10.34133/csbj.0204)
Supplement: Supplementary file 1 [file csbj.0204.f1.zip › SupplFig1.pdf]

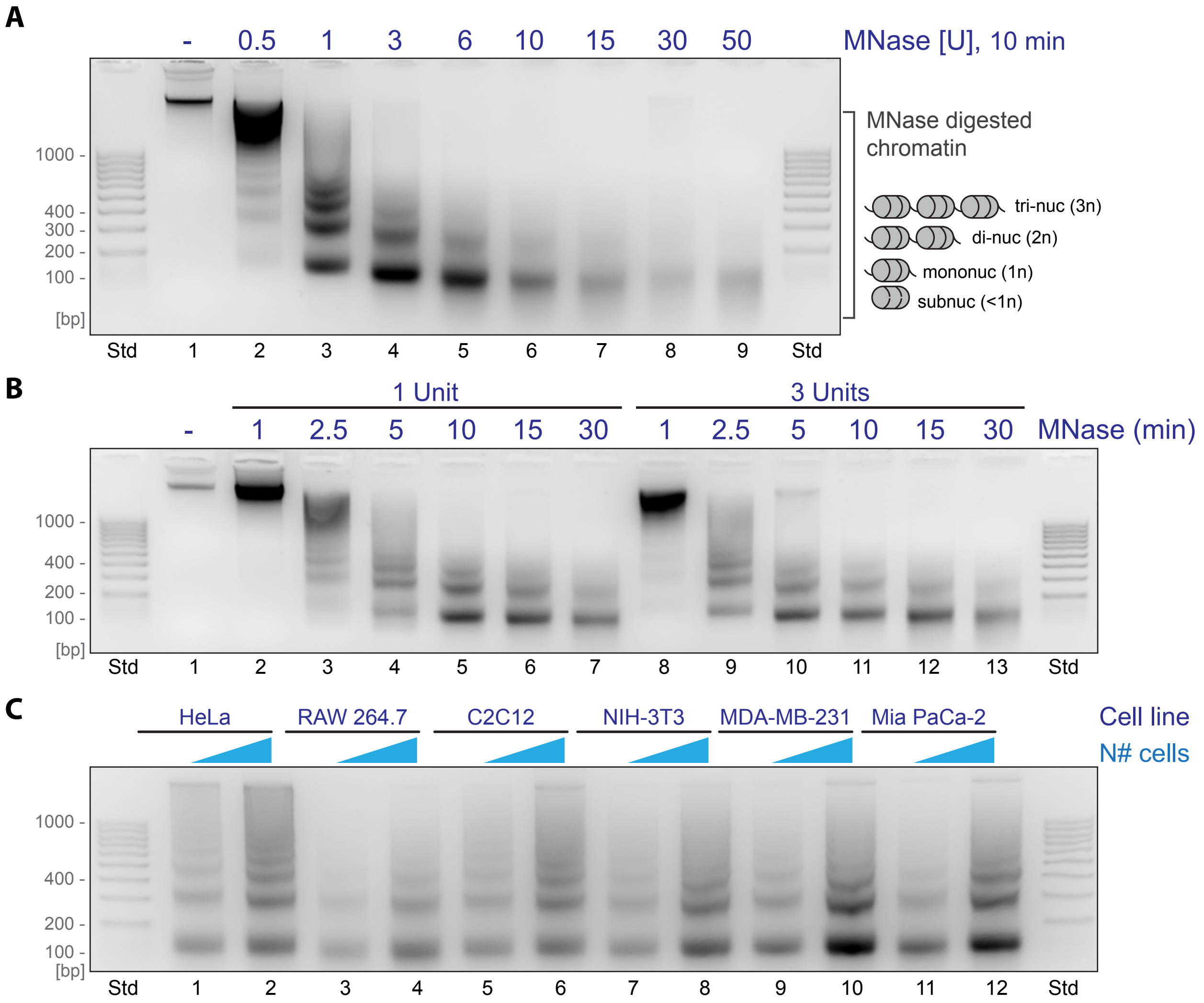

Supplementary Figure 1. MNase titration, digestion kinetics, and cross-cell-line validation of MNase-mediated chromatin digestion. (A) 2.5% agarose gel electrophoresis of MNase digestion products from a typical MNase titration experiment. The concentration-dependent MNase-digested chromatin gel displays the characteristic "nucleosome ladder" pattern, with bands corresponding to mono-nucleosomes (1n), di-nucleosomes (2n), tri-nucleosomes (3n), and longer oligonucleosome fragments. (B) 2.5% agarose gel electrophoresis of MNase digestion products comparing two MNase units (1 vs. 3) in a time-dependent experiment. The time-dependent MNase-digested chromatin exhibits the characteristic "nucleosome ladder" pattern described above. 500,000 hiPSCs were used in A and B.

(C) MNase digestion products comparing six additional mammalian cell lines commonly used in biomedical research: HeLa (cervical adenocarcinoma), RAW 264.7 (monocyte/macrophage), C2C12 (myoblast), NIH-3T3 (embryonic fibroblast), MDA-MB-231 (breast adenocarcinoma), and MIA PaCa-2 (pancreatic carcinoma). All cell lines exhibit the characteristic "nucleosome ladder" pattern following MNase digestion under the same standardized digestion conditions established for hiPSCs [3U MNase / 5 min], demonstrating that the optimized workflow generalizes across diverse cell types without further protocol modification. 250,000 (lanes 1, 3, 5, 7, 9, 11) and 500,000 (lanes 2, 4, 6, 8, 10, 12) cells of each line were used.
