# Supplementary material for: A Scalable MNase-seq Framework for Reproducible Nucleosome Profiling across Pluripotent Stem Cell and Cardiomyocyte Models
Source: Comput Struct Biotechnol J. 2026 Aug 5;35(1):0204. doi: 10.34133/csbj.0204 (PMC13438036; doi:10.34133/csbj.0204)
Supplement: Supplementary file 1 [file csbj.0204.f1.zip › SupplFig3.pdf]

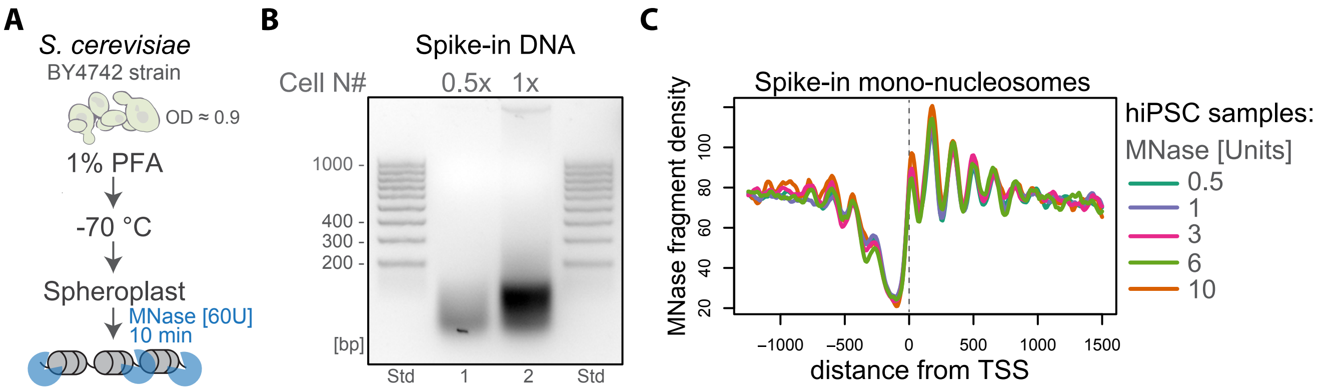

Supplementary Figure 3. Custom-generated spike-in DNA from log-phase growing yeast. (A) Schematic illustrating the in-house development of spike-in DNA from growing yeast. OD, optical density. (B) Spike-in DNA was generated using 60U of MNase for 10 minutes with two different cell quantities. The 1x cell quantity was selected for downstream spike-in analysis as it contained higher and more abundant molecular weight DNA. (C) Yeast MNase-seq DNA fragment density relative to the distance from the transcriptional start site (TSS) using increasing concentrations of MNase (0.5U to 10U), demonstrating similar footprinting patterns of spike-in mono-nucleosomes.
